# *SOD2* gene Val16Ala polymorphism is associated with macroalbuminuria in Mexican Type 2 Diabetes patients: a comparative study and meta-analysis

**DOI:** 10.1186/1471-2350-14-110

**Published:** 2013-10-11

**Authors:** Iván de Jesús Ascencio-Montiel, Esteban J Parra, Adán Valladares-Salgado, Jaime H Gómez-Zamudio, Jesús Kumate-Rodriguez, Jorge Escobedo-de-la-Peña, Miguel Cruz

**Affiliations:** 1 Biochemistry Research Unit, Hospital de Especialidades, Centro Médico Nacional “Siglo XXI”, Instituto Mexicano del Seguro Social, Mexico DF, Mexico; 2 Department of Anthropology, University of Toronto at Mississauga, Mississauga, Ontario, Canada; 3 Fundación IMSS, Mexico DF, Mexico; 4 Clinical Epidemiology Research Unit, Hospital General Regional No. 1 “Carlos MacGregor Sánchez Navarro”, Instituto Mexicano del Seguro Social, Mexico DF, Mexico

**Keywords:** Mexicans, Macroalbuminuria, SOD2, Type 2 diabetes, Val16Ala polymorphism

## Abstract

**Background:**

Several studies in type 2 diabetes patients have shown significant associations between the *SOD2* gene Val16Ala polymorphism and albuminuria, but this association has not been explored in the Mexican population.

**Methods:**

We evaluated the association between the *SOD2* gene Val16Ala polymorphism (rs4880) and macroalbuminuria in a sample of 994 unrelated Mexican type 2 diabetes patients. The study included 119 subjects with urinary albumin >300 mg/dL and 875 subjects with urinary albumin ≤ 30 mg/dL. Genotyping of the *SOD2* gene Val16Ala SNP was carried out with Real-Time Polymerase Chain Reaction (RT-PCR).

**Results:**

The frequency of the TT genotype was 6.7% higher in participants with macroalbuminuria than in the normoalbuminuria group (16.8% vs. 10.1%). Using a logistic regression analysis, we observed that individuals with the CC genotype had significantly lower risks of macroalbuminuria than those with the TT genotype (OR=0.42, p=0.034). We carried out a meta-analysis combining our data with data from four previous studies and estimated an odds ratio (95% CI) for the C allele (with respect to the reference T allele) of 0.65 (0.52-0.80, p<0.001).

**Conclusions:**

A significant association was found between the *SOD2* Val16Ala polymorphism and macroalbuminuria in a sample of Mexican type 2 diabetes patients.

## Background

Diabetic nephropathy (DN) is the leading cause of chronic kidney disease [1]. However, the risk factors for DN have not been clearly established [2] and the pathogenesis of the disease remains unclear. There is evidence indicating that oxidative stress is associated with renal damage [3]. Macroalbuminuria is a predictor of DN, cardiovascular morbidity and mortality [4-6].

Superoxide dismutase 2 (SOD2), also known as manganese superoxide dismutase (MnSOD), is one of the major antioxidant defense systems against mitochondrial superoxide radicals [7]. The Val16Ala non-synonymous polymorphism (rs4880) in the *SOD2* gene has been predicted to cause a conformational change in the target sequence, from a partial alpha-helix in the Ala-variant (coded by the C allele) to a beta-sheet in the Val-variant (coded by the T allele), which induces a 30-40% decrease in SOD2 activity due less efficient transport of the protein into the mitochondrial matrix [8,9].

Population differences in the incidence and progression of renal diseases have been described previously [10,11]. Native Americans, Hispanics and Mexicans have higher risk to develop DN in comparison with Europeans [12,13]. Contemporary Mexicans are an admixed population with genetic background derived from indigenous Mexican groups, Europeans and West Africans. For example, in a recent study in Mexico City, the relative Native American (NAM), European (EUR) and African (AFR) contributions were estimated as 65, 30 and 5%, respectively [14].

Several studies in type 2 diabetes (T2D) patients of Japanese, Korean and Chinese origin [15-18], as well as a meta-analysis [19], have shown significant associations between the *SOD2* gene Val16Ala polymorphism and albuminuria, but this association has not been explored in the Mexican population. The aim of the study was to assess the association of the *SOD2* gene Val16Ala polymorphism with macroalbuminuria in a sample of T2D patients from Mexico City.

## Methods

### Ethics statement

The project was approved by the 3609 Local Ethics Committee of the National Institute of Social Security (IMSS) according to the declaration of Helsinki. Written informed consent was obtained from each participant.

### Study group

The study included 994 unrelated Mexican T2D patients recruited from primary care clinics of the IMSS, in Mexico City, between 2008 and 2010. Diabetes diagnosis was done according to the American Diabetes Association criteria, i.e. fasting plasma glucose ≥7.0 mmol/l or OGTT ≥11.1 mmol/l for the 2 h sample [20]. Albuminuria was determined in spot urinary samples by nephelometric immunoassay. Participants with macroalbiminuria (N=119) had urinary albumin >300 mg/dL, while normoalbuminuria (N=875) was defined as urinary albumin ≤ 30 mg/dL [21]. None of the participants showed any evidence of urinary tract infection, hematuria, acute febrile illness, acute heart failure or vigorous exercise, based on clinical examination and general urine testing. Data on age, gender, duration of T2D, body mass index (BMI) and systolic and diastolic blood pressure, previous diagnosis of hypertension, and hypertension treatment with angiotensin converting enzyme (ACE) or angiotensin receptor blockers (ARB) were also available for each participant. We also measured fasting glucose, glycated hemoglobin (Hb1Ac), LDL, HDL, and total cholesterol as well as triglycerides.

### DNA isolation

DNA was extracted from peripheral blood using the QIAamp (Qiagen, Hilden, Germany) kit following the manufacturer’s recommendations. DNA concentration was measured by optical density (VICTOR3 1420 spectrophotometer Perkin-Elmer, Massachusetts, USA), and integrity by electrophoresis in 0.8% agarose gels stained with ethidium bromide (Gel Doc 2000, BIORAD, California, USA).

### *SOD2* gene Val16Ala polymorphism (rs4880) genotyping

The *SOD2* gene Val16Ala polymorphism was genotyped with RT-PCR, using the TaqMan® SNP Genotyping Assay (Applied Biosystems, California, USA).

### Ancestry estimation

Individual admixture proportions were available for 386 out of the 875 participants with normoalbuminuria, and for 53 of 119 subjects in the macroalbuminuria group. For these individuals, Native American, European and West African contributions were estimated with the program ADMIXTURE [22], using a genome-wide panel of approximately 37,000 Ancestry Informative Markers (AIMs). These 37,000 AIMs were present in both the Affymetrix Genome-Wide Human SNP array 5.0 and the Axiom LAT Genome-Wide Human Array (Affymetrix, Santa Clara, CA, USA), which were used for high-density genotyping of the samples in an unrelated study [23].

### Statistical analysis

Differences between groups were assessed using t-Student and chi-square tests for numerical and categorical variables, respectively. Deviations from the Hardy-Weinberg proportions were evaluated using an exact test (available at http://ihg.gsf.de/cgi-bin/hw/hwa1.pl).

In order to test if the *SOD2* gene Val16Ala polymorphism was associated with macroalbuminuria, we used a logistic regression model including as covariates sex, duration of T2D, body mass index, systolic blood pressure, diastolic blood pressure, current and previous smoking, HbA1c, total cholesterol, HDL cholesterol, LDL cholesterol, previous diagnosis of hypertension and hypertension treatment with ACE or ARB, which showed significant differences between groups in our preliminary univariate analyses. The effect of the *SOD2* gene Val16Ala polymorphism was tested using an unconstrained genetic model, where the odds ratios for the CC and CT genotypes were estimated independently, using the TT homozygote, which has been associated with lower SOD2 activity, as the reference genotype. In addition to the aforementioned analysis, we carried out two additional logistic regression analyses: one for the subset of the samples for which individual ancestry information was available, and another using normoalbuminuric subjects with more than 10 years of duration of T2D. The goal of these additional analyses was to determine if the association of the *SOD2* gene Val16Ala polymorphism with macroalbuminuria could be driven by population stratification (e.g. differences in ancestry between normoalbuminuria and macroalbuminuria groups) or T2D duration. Finally, an inverse variance meta-analysis including this study as well as previously published studies [15-18] was carried out with the software Epidat Version 3.1 (available at: http://www.sergas.es/MostrarContidos_N3_T01.aspx?IdPaxina=62715).

## Results

### Demographic and biochemical characteristics of study groups

A total of 119 participants with macroalbuminuria and 875 with normoalbuminuria were included in the study. Table 1 shows the clinical characteristics of participants by albuminuria group. With respect to the normoalbuminuric subjects, the group of macroalbuminuric participants included more males, had higher proportions of individuals diagnosed with hypertension and receiving hypertension treatment, had longer duration of T2D, higher systolic and diastolic blood pressure, higher HbA1c, higher total cholesterol, HDL-cholesterol and LDL-cholesterol, lower body mass index and a lower proportion of smokers. There were no significant differences in age, fasting glucose, and triglycerides between groups.

**Table 1:** Characteristics of T2D subjects by the presence of macroalbuminuria

**Characteristic**	**Macro albuminuria**	**Normo albuminuria**	***P***
n	119 (12.0)	875 (88.0)	
Male/female	73/46	271/604	<0.001
Age (years)	58.2 ± 11.2	57.2 ± 9.5	NS
Duration of T2D (years)	17.3 ± 9.8	7.2 ± 6.6	<0.001
Body mass index (kg/m^2^)	28.4 ± 4.6	29.4 ± 4.9	0.027
Systolic blood pressure (mmHg)	136.9 ± 20.2	125.7 ± 18.3	<0.001
Diastolic blood pressure (mmHg)	85.8 ± 12.1	78.8 ± 9.5	<0.001
Current or previous smoking/no smoking	38/81	418/457	0.001
Fasting glucose (mmol/L)	9.07 ± 4.43	8.8 ± 3.65	NS
HbA1c (%)	8.8 ± 3.4	7.48 ± 2.81	<0.001
Total cholesterol (mmol/L)	5.50 ± 1.86	5.12 ± 1.11	0.001
HDL cholesterol (mmol/L)	1.14 ± 0.37	1.05 ± 0.36	0.015
LDL cholesterol (mmol/L)	3.64 ± 1.4	3.15 ± 0.9	<0.001
Triglycerides (mmol/L)	2.67 ± 1.89	2.43 ± 1.74	NS
Previous diagnosis of hypertension yes/no	64/55	353/522	0.005
Hypertension treatment with ACE or ARB yes/ no	52/67	245/630	<0.001

Data is shown as number (%) or average ± standard deviation. X2 test or t-Student test P value. *NS* not significant, *ACE* Angiotensin Converting Enzyme, *ARB* Angiotensin receptor blockers.

### Association of the *SOD2* gene Val16Ala polymorphism with macroalbuminuria

**Figure 1:**
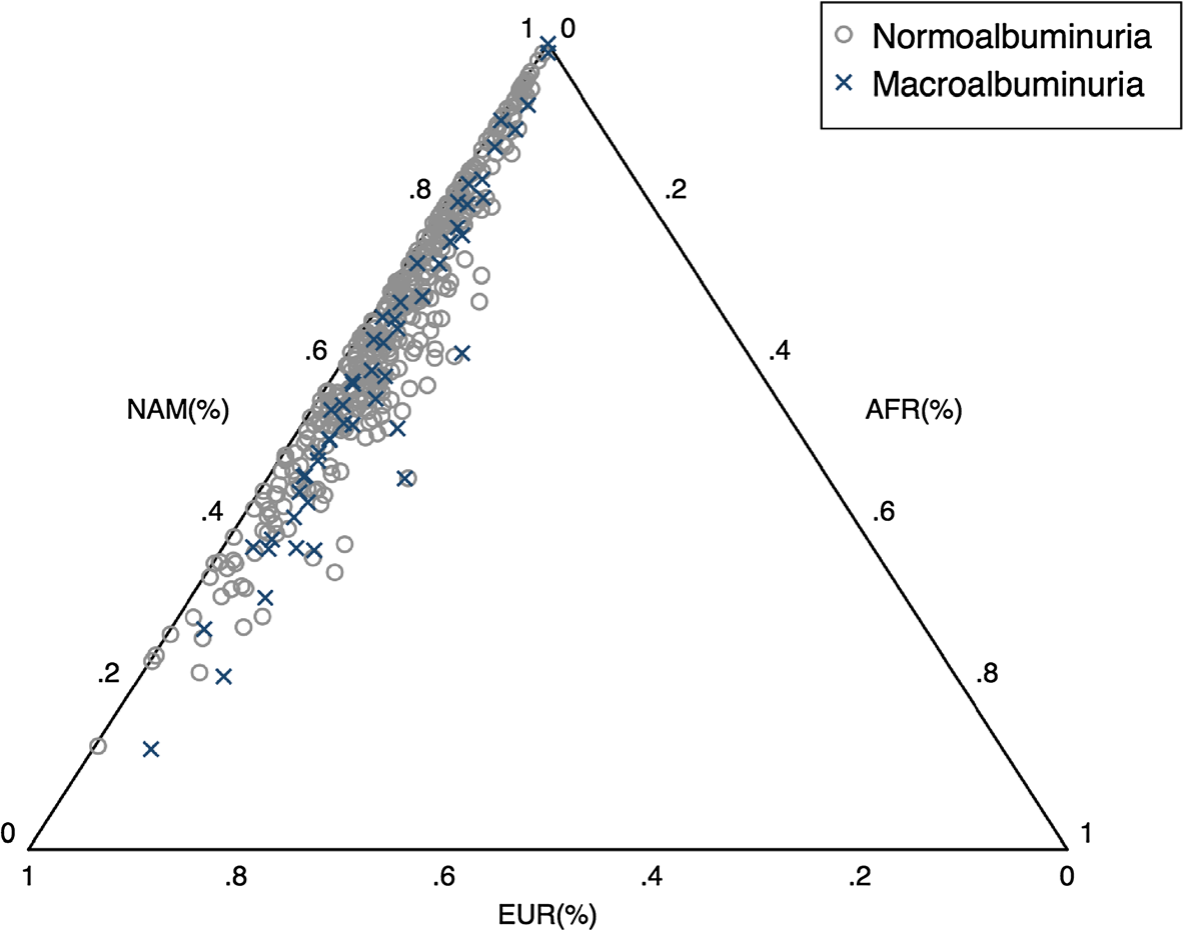
**Triangle plot of individual admixture proportions.** Triangle plot showing the distribution of the Native American (NAM), the European (EUR) and the African (AFR) individual admixture proportions, in the normoalbuminuric and macroalbuinuric subjects.

Table 2 shows the allele and genotype frequencies of the *SOD2* gene Val16Ala polymorphism in the comparison groups. There were no significant deviations from Hardy-Weinberg proportions in the normoalbuminuria or macroalbuminuria groups. The frequency of the T allele was higher in the macroalbuminuria group than in the normoalbuminuria group (41.6% vs. 32.9%). With respect to the genotypes, the frequency of the TT genotype was 6.7% higher in the participants with macroalbuminuria than in the normoalbuminuric subjects (16.8% vs. 10.1%). The effect of the *SOD2* gene Val16Ala polymorphism on macroalbuminuria was evaluated using a logistic regression model including sex, duration of T2D, body mass index, systolic blood pressure, diastolic blood pressure, current or previous smoking, HbA1c, total cholesterol, HDL-cholesterol, LDL-cholesterol, previous diagnosis of hypertension and hypertension treatment as covariates (all these variables were significantly associated with macroalbuminuria in our preliminary univariate analyses). Table 3 shows the OR estimates for all the variables showing significance in the logistic regression analysis. Using an unconstrained genetic model, the genotypes CT and CC had lower odds ratios for macroalbuminuria than the reference TT genotype (CT: OR 0.67, p=0.308; CC: OR=0.42, p=0.034). This analysis did not include in the model individual ancestry proportions, which was not available for all the participants. Restricting our attention to the subset of the samples with information on individual ancestry proportions, we observed that macroalbuminuria subjects had a lower Native American ancestry mean than normoalbuminuria group (0.597 ± 0.027 vs. 0.663 ± 0.009, p=0.012) (Figure 1). The odds ratios for the CT and CC genotypes in the logistic regression analysis incorporating Native American ancestry as a covariate were overly similar to those observed for the full sample, although the p-values are weaker due to the reduced sample size (CT: OR=0.59, p=0.400, CC: OR=0.31, p=0.081) (Figure 1). Additionally, the odds ratios for the CT and CC genotypes in the logistic regression analysis using normoalbuminuria subjects with more than 10 years of duration of T2D was quite similar (CT: OR 0.45, p= 0.077; CC: OR=0.24, p=0.002).

**Table 2:** **Distribution of the *SOD2 *gene Val16Ala polymorphism among subjects**

			**Allele**		**Genotype**			
**Gene**	**Polymorphism**	**Status**	**T**	**C**	**TT**	**CT**	**CC**	***P***_***HW***_
*SOD2*	A16V	Macroalbuminuria	99(41.6)	139(58.4)	20(16.8)	59(49.6)	40(33.6)	0.650
	C>T	Normoalbuminuria	576(32.9)	1174(67.1)	88(10.1)	400(45.7)	387(44.2)	0.320

P_HW_: Hardy-Weinberg equilibrium exact P value. Data is shown as number (%).

**Table 3:** **Results of the logistic regression analysis of the *SOD2 *gene Val16Ala polymorphism and macroalbuminuria**

**Variable**	**OR (95%CI)**	***P***
Male sex	4.26 (2.41-7.53)	<0.001
Duration of T2D (years)	1.14 (1.10-1.17)	<0.001
Diastolic blood pressure (mmHg)	1.06 (1.03-1.09)	<0.001
Current or previous smoking	0.41 (0.23-0.71)	0.002
HbA1c (%)	1.15 (1.05-1.25)	0.002
LDL cholesterol (mmol/L)	1.99 (1.29-3.10)	0.002
rs4880		
CT genotype^1^	0.67 (0.31-1.45)	NS
CC genotype^1^	0.42 (0.18-0.93)	0.034

Logistic regression analysis including sex, duration of T2D, body mass index, systolic blood pressure, diastolic blood pressure, current and previous smoking, HbA1c, total cholesterol, HDL cholesterol, LDL cholesterol, previous diagnosis of hypertension, hypertension treatment with ACE or ARB, and the *SOD2* gene Val16Ala polymorphism genotype. ^1^TT is the reference genotype.

### Meta-analysis with other available studies

**Figure 2:**
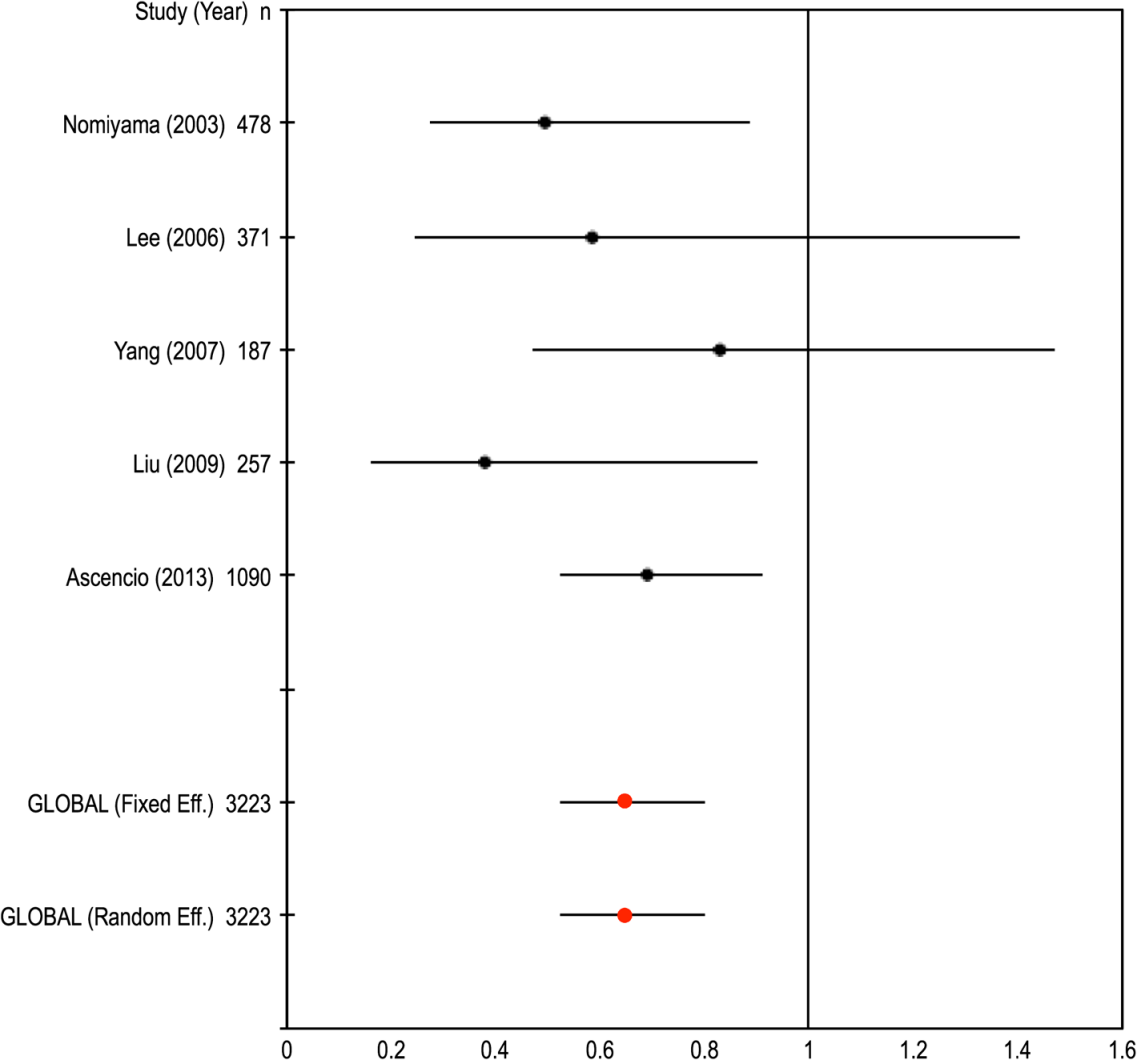
**Forest plot of macroalbuminuria and *SOD2 *gene Val16Ala polymorphism.** Forest plot of ORs for macroalbuminuria and *SOD2* gene Val16Ala polymorphism in dominant model (CC+TC vs TT) in studies with T2D patients.

We carried out an inverse variance meta-analysis including our study and four additional studies for which genotype data are available. We report the results using an allele model (C vs. T), but other models (e.g. codominant or dominant TT vs. CC/CT) give consistent results. Table 4 reports the genotype and allele frequencies in the comparison groups, odds ratios and corresponding p-values for each study. There was not evidence of significant heterogeneity between studies (Q=3.28, p=0.512), so we report here the results of the fixed effects meta-analysis. A forest plot with the estimates of odds ratios and 95% confidence intervals (CI) for individual studies, as well as the combined odds ratios and CI based on fixed effects and random effects models, is shown in Figure 2. The results of the meta-analysis are significant, with an odds ratio (CI) of 0.65 (0.52-0.80, p<0.001) for the fixed effects model. A sensitivity analysis excluding one study at a time indicates that the results of the meta-analysis are quite robust, with estimates of the odds ratios ranging from 0.52 to 0.80).

**Table 4:** **Distribution of the *SOD2 *gene Val16Ala polymorphism among groups, in albuminuria studies with T2D patients**

			**Genotype TT/CT/CC**				**C allele frequency**			
			**Macroalbuminuria**		**Normoalbuminuria**					
**Authors (reference)**	**Year**	**Population**	**Number**	***P***_**HW**_	**Number**	***P***_**HW**_	**Cases**	**Controls**	**OR (95%CI)**	***P***
Nomiyama (15)	2003	Japan	74/14/0	1.000	206/83/2	0.038	8.0	14.9	0.49 (0.27-0.89)	0.017
Lee (16)	2006	Korean	36/4/1	0.178	203/24/17	<0.001	7.3	11.9	0.59 (0.24-1.40)	0.225
Yang (17)	2007	Chinese	47/15/11	1.000	23/25/2	0.183	25.3	29.0	0.83 (0.47-1.47)	0.525
Liu (18)	2009	Chinese	55/7/0	1.000	78/22/3	0.390	5.6	13.6	0.38 (0.16-0.90)	0.023
Ascencio	2013	Mexican	20/59/40	1.000	88/400/387	0.320	58.4	63.7	0.69 (0.52-0.91)	0.008
All studies			232/99/52	<0.001	598/554/411	<0.001	26.5	44.0	0.65 (0.52-0.80)	<0.001

*P*_*HW*_ Hardy-Weinberg equilibrium P value, *OR* odds ratio, *CI* confidence interval, *P* P value between macroalbuminuric and normoalbuminuric subjects.

## Discussion

Diabetic nephropathy is an important public health problem in Mexico. DN is the leading cause of end stage renal disease in adults and is responsible for approximately 75% of the total costs associated with the treatment of diabetic complications [24]. Previous research has indicated that there are population differences in the risk of developing end stage renal disease, which is significantly higher in African Americans, Asians and Latinos, relative to individuals of European ancestry [10]. However, the ultimate reasons for these prevalence differences have not been fully elucidated. Many hypotheses have been proposed to explain the development of diabetes complications, including DN. One of the hypotheses is that oxidative stress plays a major role in the pathogenesis of diabetes complications [25]. For this reason, it is important to explore the potential effect of polymorphisms located within the genes coding for antioxidant enzymes, such as the *SOD2* gene, on DN risk. A number of studies have focused on the *SOD2* gene Val16Ala polymorphism, which is a non-synonymous polymorphism that has been associated with DN in several populations. However, although polymorphisms in the ACE [26] and TGF-β [27] genes have been previously associated with DN in the Mexican population, to our knowledge, the association between the *SOD2* gene Val16Ala polymorphism and macroalbuminuria has not been yet studied in Mexico.

We genotyped the *SOD2* gene Val16Ala polymorphism in 994 T2D patients: 119 individuals with macroalbuminuria and 875 normoalbuminuric subjects. In a logistic regression model incorporating other relevant covariates, the CT and CC genotypes had lower odds ratios than the reference TT genotype (CT: OR 0.67, p=0.308; CC: OR=0.42, p=0.034). Given the different odd ratios observed for the CT and CC genotypes, a codominant model (CC vs. CT vs. TT) seems to provide the best fit to the data. Using a dominant model, instead of an unconstrained model, the CC+CT genotypes had an OR=0.55 (p=0.026), with respect to the TT genotype.

To our knowledge, ours is the largest study to date evaluating the association of the *SOD2* gene Val16Ala polymorphism with macroalbuminuria. Our results are consistent with a growing number of studies in different population groups [15-19], and combining our study with previously available data provides strong support for the hypothesis that the *SOD2* Val16Ala C allele, which codes for the amino acid alanine, confers a protective effect against macroalbuminuria. This protective effect has been explained as a result of the conformational change caused by alanine, which increases the efficiency of protein targeting to the mitochondria, thereby leading to a rise of reactive oxygen species scavenging in the mitochondria, and a reduction in glomerular membrane damage due to oxidative stress [8,9].

The contemporary Mexican population is the result of an admixture process that involved Native American, European and African populations. It is well known that the presence of population stratification can inflate the rate of false positives in association studies in admixed populations [28]. For example, when there are substantial differences in admixture proportions between cases and controls, markers showing large frequency differences between the parental populations may be significantly associated with the disease, even if they do not play any causal role. One way to correct for the effects of population stratification is to include individual admixture estimates in the statistical models. Unfortunately, we do not have ancestry estimates for all the individuals included in this sample. However, this information is available for approximately half of the sample, and this gave us the opportunity to explore if the inclusion of admixture estimates in the model may alter the conclusions of our study.

When we restricted our analysis to the participants for which admixture information was available, we observed that macroalbuminuria subjects had a lower Native American ancestry than normoalbuminuria group (0.597 ± 0.027 vs. 0.663 ± 0.009, p=0.012). The odds ratios of the logistic regression analysis incorporating ancestry in the model are similar to those observed in the full sample, again indicating that the CC and CT genotypes have a protective effect against macroalbuminuria (CT: OR=0.59, p=0.400, CC: OR=0.31, p=0.081). The p-values obtained in this analysis are slightly weaker than in the full sample, but this is not surprising considering that the sample size was reduced in half, with a subsequent reduction in power to identify significant effects. Overall, our data suggests that the significant results observed in the analysis of the full dataset are not due to the presence of population stratification. If stratification was responsible for the results observed in the full sample, we would expect that an analysis of the subsample incorporating ancestry in the statistical models would lead to differences in the ORs observed in both samples. Instead, the ORs are similar in both analyses (in fact, the ORs for the CC and CT genotypes are slightly lower in the model including ancestry than those observed in the full sample, without correction for variation in ancestral proportions). We also estimated the odds ratios restricting the normoalbuminuric sample to subjects with more than 10 years of duration of T2D and the results are very similar to those observed in the full sample.

The allelic frequency of the *SOD2* Val16Ala polymorphism risk allele T, which codes for the amino acid valine, in the normoalbuminuric group from Mexico City is quite similar to the frequencies observed in the HapMap Mexican American sample from Los Angeles, California (32.9% vs. 36.0%) (http://hapmap.ncbi.nlm.nih.gov/cgi-perl/snp_details_phase3?name=rs4880&source=hapmap28_B36&tmpl=snp_details_phase3). However, the data available for other population groups indicate that there is substantial dispersion of allele frequencies for this polymorphism. For example, in populations from East Asia the frequencies of the T allele are higher than 85%, and in populations of African and European ancestry, the T allele has intermediate frequencies, between 50% and 65%.

In our logistic regression analyses, we included a number of covariates that were significantly associated with macroalbuminuria in our sample (sex, duration of T2D, body mass index, systolic blood pressure, diastolic blood pressure, current or previous smoking, HbA1c, total cholesterol, HDL-cholesterol, LDL-cholesterol, previous diagnosis of hypertension and hypertension treatment). We observed that adding the *SOD2* Val16Ala polymorphism to a model including the aforementioned factors increased only slightly the goodness of fit of the model (McFadden's pseudo R2 values increased from 0.361 to 0.369). Some of the factors identified in our study (male sex, duration of T2D, diastolic blood pressure, HbA1c and LDL cholesterol levels) have also been highlighted in previous studies [29,30]. However, in our sample we found that current or previous smoking was inversely associated with macroalbuminuria. This stands in contrast to other studies, in which smoking was found to be a risk factor for DN [29-32], although other studies failed to find associations of smoking and DN [33,34]. Unfortunately, we only had information about current or previous tobacco use, and data were not available about duration or intensity of smoking. Other limitations of the study include the lack of information about other microvascular complications such as retinopathy or neuropathy, as well as for other factors that may be important in the pathogenesis of macroalbuminuria, such as practice of regular physical activity, type of diabetes medication, or variation in other enzymatic and non-enzymatic antioxidants. Finally, it is important to note that the sample of macroalbuminuria patients was relatively small (119 individuals). This is related to the low prevalence of macroalbuminuria among T2D patients. For example, De Pablos et al. have reported that the prevalence of macroalbuminuria among T2D patients in a Spanish population was approximately 12% [35], and in Mexico the prevalence has been estimated between 9 and 10% [36,37].

## Conclusions

In summary, we show that the *SOD2* gene Val16Ala polymorphism is associated with macroalbuminuria. Our results highlight the importance of exploring the effects of polymorphisms located within genes relevant to oxidative stress in order to understand the genetic basis of diabetes complications.

## Abbreviations

ACE: Angiotensin converting enzyme; AFR: African; AIMs: Ancestry Informative Markers; ARB: Angiotensin receptor blockers; CI: Confidence intervals; DN: Diabetic nephropathy; EUR: European; Hb1Ac: Glycated hemoglobin; IMSS: National Institute of Social Security; MnSOD: Manganese superoxide dismutase; NAM: Native American; RT-PCR: Real-time polymerase chain reaction; SOD2: Superoxide dismutase 2; T2D: Type 2 diabetes.

## Competing interests

The authors declare that they have no competing interests.

## Authors’ contributions

IJAM, EJP, AVS, JKR and MC conceived and designed the study. IJAM, AVS, JEP and JHGZ acquired the data. IJAM, EJP, AVS, MC interpreted the data. All authors revised the manuscript for important intellectual content, read, and approved the final manuscript.

## Pre-publication history

The pre-publication history for this paper can be accessed here:

http://www.biomedcentral.com/1471-2350/14/110/prepub
